# Non-Pharmacological Treatments for Oral Carcinogenesis: From Oral Lichen Planus to Oral Squamous Cell Carcinoma

**DOI:** 10.3390/cancers18182949

**Published:** 2026-09-11

**Authors:** Federica Re, Francesco Scilla, Mauro Labanca, Domenico Russo, Gioele Gioco, Gaia Favero, Rita Rezzani

**Affiliations:** 1 Department of Clinical and Experimental Sciences, Università di Brescia, 25123 Brescia, Italy; federica.re@unibs.it (F.R.); mauro.labanca@unibs.it (M.L.); gaia.favero@unibs.it (G.F.); 2 Unit of Blood Diseases and Bone Marrow Transplant, Department of Clinical and Experimental Sciences, Università di Brescia, ASST Spedali Civili, 25123 Brescia, Italy; domenico.russo@unibs.it; 3 Interdepartmental University Center of Research “Adaption and Regeneration of Tissues and Organs (ARTO)”, Università di Brescia, 25123 Brescia, Italy; francesco.scilla@unicatt.it; 4 Head and Neck Department, Fondazione Policlinico Universitario A. Gemelli-IRCCS, School of Dentistry, Università Cattolica del Sacro Cuore, 00168 Rome, Italy; gioele.gioco@unicatt.it; 5 Italian Society for the Study of Orofacial Pain (Società Italiana Studio Dolore Orofacciale—SISDO), 25123 Brescia, Italy

**Keywords:** oral lichen planus, oral squamous cell carcinoma, oral potentially malignant disorders, non-pharmacological therapies, oral carcinogenesis, photobiomodulation, photodynamic therapy, nutraceuticals, oral hygiene management

## Abstract

Oral lichen planus (OLP) is a chronic, immune-mediated inflammatory disorder of the oral mucosa recognized as an oral potentially malignant disorder, with a pooled malignant transformation rate of approximately 1–1.4% that is highest in atrophic-erosive and tongue lesions. Its most feared outcome, oral squamous cell carcinoma (OSCC), remains associated with a 5-year survival below 50% and a substantial clinical, functional, and economic burden. Because conventional management, like topical corticosteroids for OLP and surgery with or without radiotherapy for OSCC, entails cumulative toxicity and limited long-term efficacy, non-pharmacological strategies are attracting growing interest. This narrative review critically appraises the available evidence across the OLP-to-OSCC continuum, examining high-power ablative lasers, low-level laser therapy/photobiomodulation, photodynamic therapy, nutraceutical and phytotherapeutic agents (including melatonin, curcumin, aloe vera, coconut, zinc, and vitamin D), cryotherapy, ozone therapy, probiotics, and psychological interventions, alongside supportive measures in OSCC such as oral hygiene management, photobiomodulation for treatment-related toxicity, and post-treatment rehabilitation. Evidence is strongest and most consistent for photobiomodulation in OLP, whereas most other modalities remain promising but limited by small samples, heterogeneous protocols, and scarce placebo-controlled data. A multidisciplinary, evidence-graded approach is essential to optimize early detection, disease control, and quality of life.

## Simple Summary

Oral lichen planus (OLP) is a chronic T-cell-mediated inflammatory disorder of the oral mucosa, affecting approximately 1–2% of adults. It is classified by the World Health Organization as an oral potentially malignant disorder, with a malignant transformation rate of about 1%, particularly in atrophic and erosive lesions, tongue involvement, and in the presence of risk factors such as tobacco, alcohol, and hepatitis C infection. Oral squamous cell carcinoma (OSCC), the most common oral malignancy, remains associated with high morbidity and mortality despite advances in treatment. Conventional management relies on topical corticosteroids for OLP and surgery with or without radiotherapy for OSCC, but these approaches are limited by side effects, recurrence, and functional impairment. This review critically examines non-pharmacological strategies across the OLP–OSCC continuum, including laser and light-based therapies, photodynamic therapy, nutraceuticals, ozone therapy, probiotics, oral hygiene, and rehabilitation, highlighting their evidence, safety, and potential role in improving patient outcomes and quality of life.

## 1. Introduction

Oral lichen planus (OLP) is a chronic, immune-mediated inflammatory disease of the oral mucosa, considered to result from a T-cell-mediated cytotoxic reaction directed against basal keratinocytes, although its precise etiology remains incompletely understood [1]. It affects approximately 1–2% of the general population, with a female predilection and a mean age of onset in the fourth to sixth decade of life [2,3]. Clinically, OLP is classified into six recognized subtypes: reticular, papular, plaque-like, atrophic, erosive, and bullous. The reticular form, characterized by lace-like white striae (Wickham’s striae) typically distributed bilaterally on the buccal mucosa, is the most common and least symptomatic, whereas the erosive and atrophic variants present with erythema, ulceration, and pain, and are associated with greater diagnostic and therapeutic complexity [3,4].

The diagnosis of OLP rests on the integration of clinical examination with histopathological confirmation. The original World Health Organization (WHO) criteria proposed in 1978 were subsequently revised by van der Meij and van der Waal in 2003 to improve reproducibility, retaining a well-defined band-like subepithelial lymphocytic infiltrate and liquefaction degeneration of the basal cell layer as essential features [5]. These modified WHO criteria remain the most widely applied diagnostic standard, although more recent proposals, including the 2016 criteria of the American Academy of Oral and Maxillofacial Pathology, have highlighted persistent discrepancies in histopathological interpretation and a risk of underdiagnosing lesions with early dysplastic change [6]. Management of OLP is primarily aimed at controlling symptoms, particularly for the erosive and atrophic subtypes, and consists predominantly of topical corticosteroids (e.g., triamcinolone acetonide or clobetasol propionate) as first-line therapy, with topical calcineurin inhibitors reserved for refractory or steroid-intolerant cases. Non-pharmacological measures such as smoking cessation, alcohol avoidance, optimization of oral hygiene, and dietary modification (avoidance of spicy, acidic, or abrasive foods) are also recommended as adjuncts, alongside regular clinical follow-up given the chronic, relapsing-remitting course of the disease [7,8].

This last point underscores the clinical relevance of OLP beyond symptom control; in particular, OLP is currently classified by the WHO as an oral potentially malignant disorder (OPMD), defined as “any oral mucosal abnormality associated with a statistically increased risk of developing cancer” [9,10]. Meta-analytic data indicate a pooled malignant transformation rate for OLP in the range of approximately 1–1.4% [11], lower than earlier historical estimates, with transformation risk shown to vary according to the diagnostic criteria applied, being consistently lower when the strict 2003 modified WHO criteria are used [12,13]. Erythematous/erosive and atrophic subtypes, tobacco and alcohol use, and hepatitis C seropositivity have been identified as factors associated with a higher likelihood of transformation [12,14]. This oncogenic potential places OLP on the broader clinical continuum of oral potentially malignant disorders that precede the development of oral squamous cell carcinoma (OSCC)—the most frequent malignancy of the oral cavity, accounting for the large majority of head and neck cancers [15]. OLP is only one of several OPMDs; others, notably oral leukoplakia, erythroplakia, and oral submucous fibrosis, generally carry a higher risk of malignant transformation. A recent large database analysis reported 5-year malignant transformation rates of 0.2% for OLP, 8.4% for oral leukoplakia, and 50.0% for erythroplakia. Unlike these predominantly epithelial, carcinogen-driven lesions, OLP is a chronic T-cell–mediated immune-inflammatory disorder, and it is precisely this active immunological and oxidative-stress microenvironment that non-pharmacological, immunomodulatory therapies are best positioned to target. For this reason, together with the chronic, relapsing course of OLP and the consequent need to limit cumulative corticosteroid exposure, the present review deliberately focuses on the OLP-to-OSCC axis; a comprehensive discussion of the other OPMDs lies beyond its scope. OSCC accounts for approximately 90% of all oral cancers and affects the lips, tongue, floor of the mouth, buccal mucosa, gingiva, hard palate, and retromolar trigone, with the tongue being the most frequently involved site. About 389,500 new cases and 188,200 deaths were reported globally in 2022 [16,17]. The total 5-year survival rate is still less than 50% despite improvements in diagnosis and treatment [18]. OSCC can have a poor prognosis; it spreads by forming metastases primarily via the lymphatic system to the lymph nodes in the neck. However, metastatic spread can also occur via the bloodstream, particularly to the lungs. For oral cancer, the 5-year survival rate from diagnosis is 44% overall, and it is higher for women (54%) than for men (41%). Although extremely invasive and debilitating, the treatment is generally not very effective in the medium- to long-term, given that approximately 80% of patients with stage III and IV disease die within the first 5 years; furthermore, after primary treatment with surgery and/or radiation therapy, recurrences or metastases occur in more than 50% of patients (most o within the first 2 years), and the 5-year survival rate in these cases is less than 50%. Early diagnosis has been shown to be a factor that leads to a better prognosis, as it results in a 5-year survival rate of approximately 80–90% (for stage I and II tumors), in addition to significantly reducing the cost of treatment.

Beyond its clinical impact, OSCC imposes a substantial economic burden on healthcare systems and patients alike [19]. Direct healthcare costs per patient are strongly stage-dependent, with data from a tertiary referral center in Italy indicating an average expenditure of approximately EUR 26,000 per patient overall, rising from roughly EUR 10,700 for stage I disease to nearly EUR 40,000 for stage IV, reflecting the cumulative cost of extended surgical hospitalization, microvascular reconstruction, and adjuvant chemoradiotherapy required in advanced cases [20]. Indirect costs, including productivity loss, informal caregiving, and long-term rehabilitation, may further exceed direct medical expenditure, reinforcing the broader societal case for strategies that enable earlier diagnosis and reduce the risk of progression from potentially malignant disorders such as OLP to invasive carcinoma [19].

Various indicators of oral hygiene have been linked to an increased risk of developing OSCC, including the presence of plaque and tartar, dental caries, or tooth loss. It is important to note, however, that poor personal hygiene often coexists with other risk factors [21], including population-associated environmental and socioeconomic conditions, as well as alcohol and tobacco use.

It is therefore difficult to establish the primary role played by poor hygiene; however, it can be hypothesized that chronic irritation of the mucous membranes, caused by poor hygiene, may modify or initiate mechanisms of neoplasm development or promote and amplify the effects of other risk factors [21].

Given the chronic and potentially premalignant nature of OLP, and the considerable disease and treatment burden of OSCC, there has been growing interest in non-pharmacological therapeutic strategies as a complement to conventional pharmacological approaches, particularly in patients who would otherwise require prolonged or repeated courses of topical or systemic medication, with the aim of reducing cumulative drug exposure and associated side effects while maintaining adequate disease control. Among these, light-based therapies—including low-level laser therapy/photobiomodulation (PBM) and photodynamic therapy (PDT)—have attracted particular attention for their reported anti-inflammatory, analgesic, and potentially antitumor effects, although their use in patients with a premalignant or malignant oral lesion remains debated in light of theoretical concerns regarding stimulation of epithelial proliferation [22,23].

The aim of the present narrative review is therefore to critically examine the current evidence on non-pharmacological therapeutic modalities employed in the management of OLP and OSCC, with particular attention to their rationale, reported efficacy, and safety profile.

## 2. Materials and Methods

### 2.1. Search Strategy

This narrative review was conducted in accordance with the principles of narrative review methodology for synthesizing evidence on complex, multifaceted clinical topics. Electronic searches were performed in PubMed/MEDLINE, Scopus, Web of Science, the Cochrane Library, and Embase, without date restriction, up to July 2025. Searches were structured around the following core terms, combined using Boolean operators: (“oral lichen planus” OR “OLP”) AND (“non-pharmacological” OR “laser therapy” OR “photobiomodulation” (PBM) OR “low-level laser” (LLLT) OR “photodynamic therapy” (PTT) OR “curcumin” OR “aloe vera” OR “nutraceutical” OR “phytotherapy” OR “ozone” OR “cryotherapy” OR “probiotic” OR “psychological” OR “behavioral”); and (“oral squamous cell carcinoma” OR “OSCC” OR “oral cancer”) AND (“photobiomodulation” OR “oral hygiene” OR “rehabilitation” OR “supportive care” OR “surgery” OR “radiotherapy”). Additional records were identified through manual screening of the reference lists of included systematic reviews and meta-analyses. No language restriction was applied, although only articles with full English text or an English abstract were retained for data extraction. Gray literature, conference abstracts not associated with a peer-reviewed publication, and unpublished data were excluded.

### 2.2. Eligibility Criteria and Study Selection

**Figure 1:**
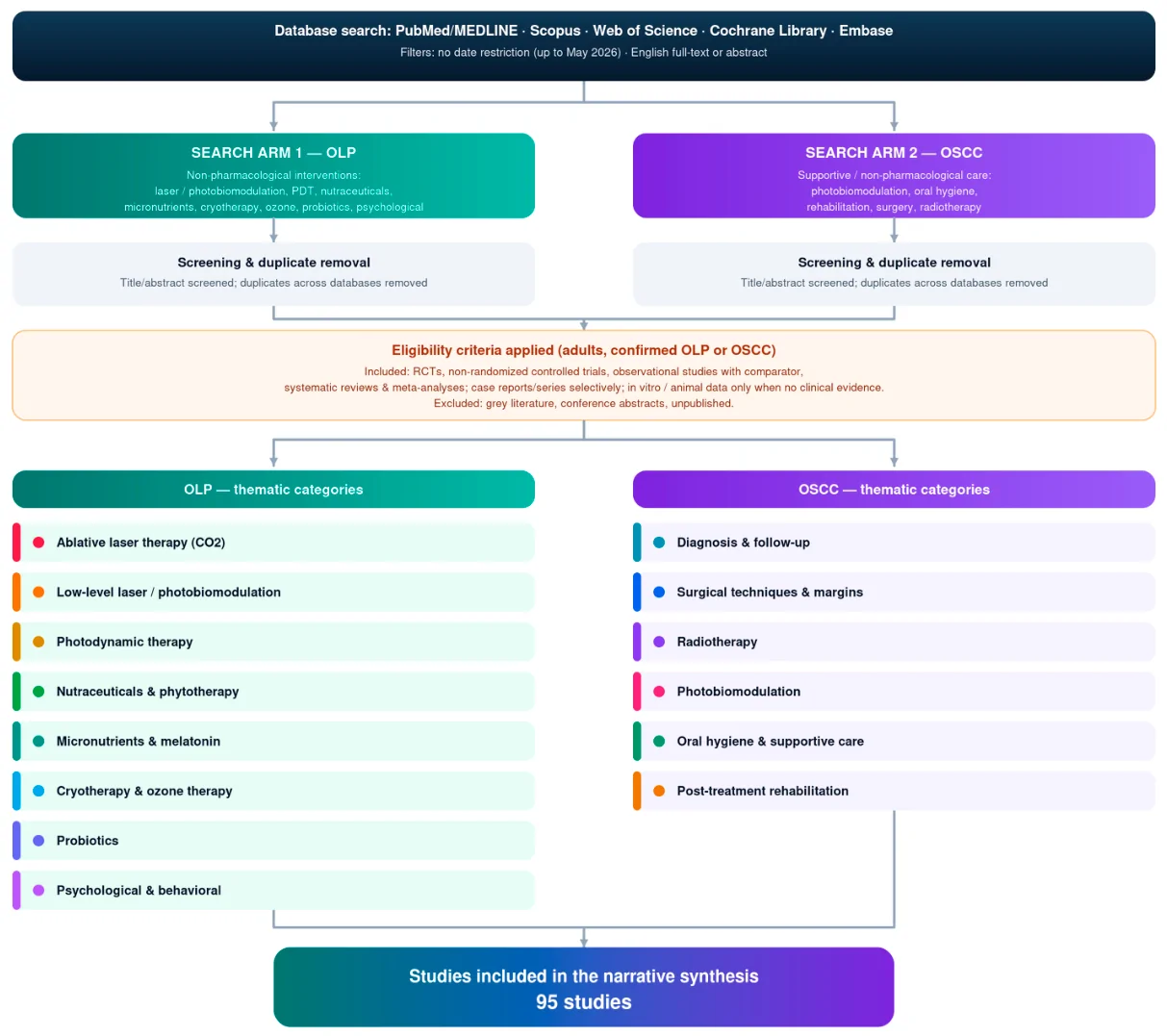
Literature search and study selection workflow. Created with Inkscape (v. 1.4.3).

Studies were eligible for inclusion if they reported original clinical data or synthesized evidence on non-pharmacological interventions in adult patients with a histologically confirmed or clinically established diagnosis of OLP or OSCC. Eligible study designs included randomized controlled trials (RCTs), non-randomized controlled clinical trials, observational studies with a comparator group, systematic reviews, and meta-analyses; case reports and uncontrolled case series were included selectively when they provided relevant mechanistic or pioneering clinical evidence that was not available from controlled studies. Studies reporting exclusively in vitro or animal data were considered only when no clinical evidence was available for a given treatment modality and are presented as such. As a narrative review, this work does not aim to pool quantitative data and is not registered in PROSPERO; nonetheless, to ensure transparency and reproducibility, the identification, screening, and inclusion of the sources are summarized in a PRISMA-style flow diagram (Figure 1). Study selection was guided by clinical relevance, study design quality, and representativeness of the available evidence base for each intervention category. When systematic reviews or meta-analyses were available, these were prioritized over primary studies for efficacy and safety summaries; primary studies are discussed directly when they contribute data not captured in existing synthesized evidence, or when they postdate the most recent relevant review.

## 3. Non-Pharmacological Therapies for Oral Lichen Planus

### 3.1. High-Power Ablative Laser Therapy

In relation to OLP therapies, high-power ablative lasers (HPALT), including carbon dioxide (CO_2_), erbium-doped yttrium aluminum garnet (Er:YAG), and neodymium-doped yttrium aluminum garnet (Nd:YAG) systems, act primarily through water absorption within the target tissue, enabling precise vaporization or excision of lesions related to OLP. A systematic review reported significant intragroup reductions in lesion size and pain following CO_2_ laser therapy, with laser treatment outperforming pharmacological comparators in studies that included a drug-comparison arm [24].

A more recent systematic review of high-power laser therapy (HPLT) that included eight studies confirmed significant reductions in lesion size, pain, and recurrence rates compared with conventional treatment, with CO_2_ lasers showing superior outcomes in lesion resolution and pain relief and Er:YAG lasers offering advantages in precision for localized lesions; reductions in inflammatory biomarkers such as interleukin-1β (IL-1β), interleukin-6 (IL-6), and interferon-γ (IFN-γ) accompanied clinical improvement in the single study that assessed them [24]. An RCT directly comparing CO_2_ laser vaporization with intralesional triamcinolone acetonide found significantly greater reductions in the reticulation/keratosis, erythema, and ulceration (REU) score, Thongprasom sign score, visual analog scale (VAS) pain score, and lesion area on the laser-treated side [25]. Long-term data from a retrospective cohort of 21 patients with 39 lesions treated with CO_2_ laser evaporation reported that 85% of patients remained pain-free over a mean follow-up of 8 years, with the remaining 15% experiencing painful recurrence that responded to repeat laser treatment [26].

Collectively, these findings support HPLT as an effective option for symptomatic, treatment-resistant OLP, particularly for erosive and atrophic lesions.

### 3.2. Low-Level Laser Therapy and Photobiomodulation

LLLT and PBM exert their effects through non-ablative mechanisms, including reduced synthesis of pro-inflammatory mediators, enhanced mitochondrial activity, analgesic effects, and promotion of tissue repair, typically using wavelengths in the 600–1100 nm range. Their proposed mechanisms are intentionally non-ablative; for example, mitochondrial cytochrome c oxidase activation increases ATP production, pro-inflammatory cytokines (tumor necrosis factor-α—TNF-α, IL-1β, IL-6) are downregulated, microcirculation is enhanced, and nerve conduction is modulated, together producing analgesic, anti-inflammatory, and pro-regenerative effects without thermal tissue damage [27,28].

Comparative randomized trials of topical corticosteroids have produced evidence that, while occasionally discordant in earlier and smaller studies, increasingly favors PBM. A recent randomized, single-blind trial comparing 660 nm PBM with 0.1% triamcinolone acetonide in 60 patients with symptomatic OLP reported significantly greater reductions in pain and lesion severity in the PBM arm at multiple follow-up points, together with a higher proportion of patients achieving clinically meaningful lesion regression and a complete absence of adverse events, in contrast to the mild local irritation observed in the corticosteroid group [27]. Comparable findings of equivalence or superiority emerge from other randomized trials, including one correlating clinical improvement with a parallel reduction in salivary oxidative stress markers, suggesting that PBM’s benefit may extend beyond symptom palliation to measurable biochemical modulation of the inflammatory microenvironment [28].

This rationale is reinforced by molecular-level evidence. A recent immunohistochemical study demonstrated that PBM with an 810 nm diode laser significantly restored expression of the anti-apoptotic marker Bcl-2 and the proliferative marker Ki-67 in OLP lesions, both of which are depressed relative to healthy mucosa, suggesting that PBM may act not only symptomatically but also by directly counteracting the keratinocyte apoptosis that characterizes OLP pathogenesis; notably, erosive forms showed the most favorable histological and clinical response [29].

At the level of synthesized evidence, conclusions have evolved considerably over the past decade. Early systematic reviews described the comparative efficacy of LLLT as “debatable,” reflecting small sample sizes, inconsistent laser parameters, and a generally high risk of bias [30]. More recent, methodologically stronger meta-analyses have converged on more consistent conclusions; for instance, PBM achieves analgesic efficacy comparable to topical corticosteroids, with superior outcomes when total energy density remains below 120 J/cm^2^ or when more than ten sessions are administered, whereas high-intensity laser therapy may provide even greater short-term pain relief at the cost of a narrower safety margin [31]. Longer-term analyses further indicate that PBM reduces recurrence rates relative to corticosteroid therapy, with benefit sustained up to 12 weeks after treatment completion, alongside a consistently favorable safety profile across studies, with virtually no adverse events directly attributable to PBM [31,32].

**Figure 2:**
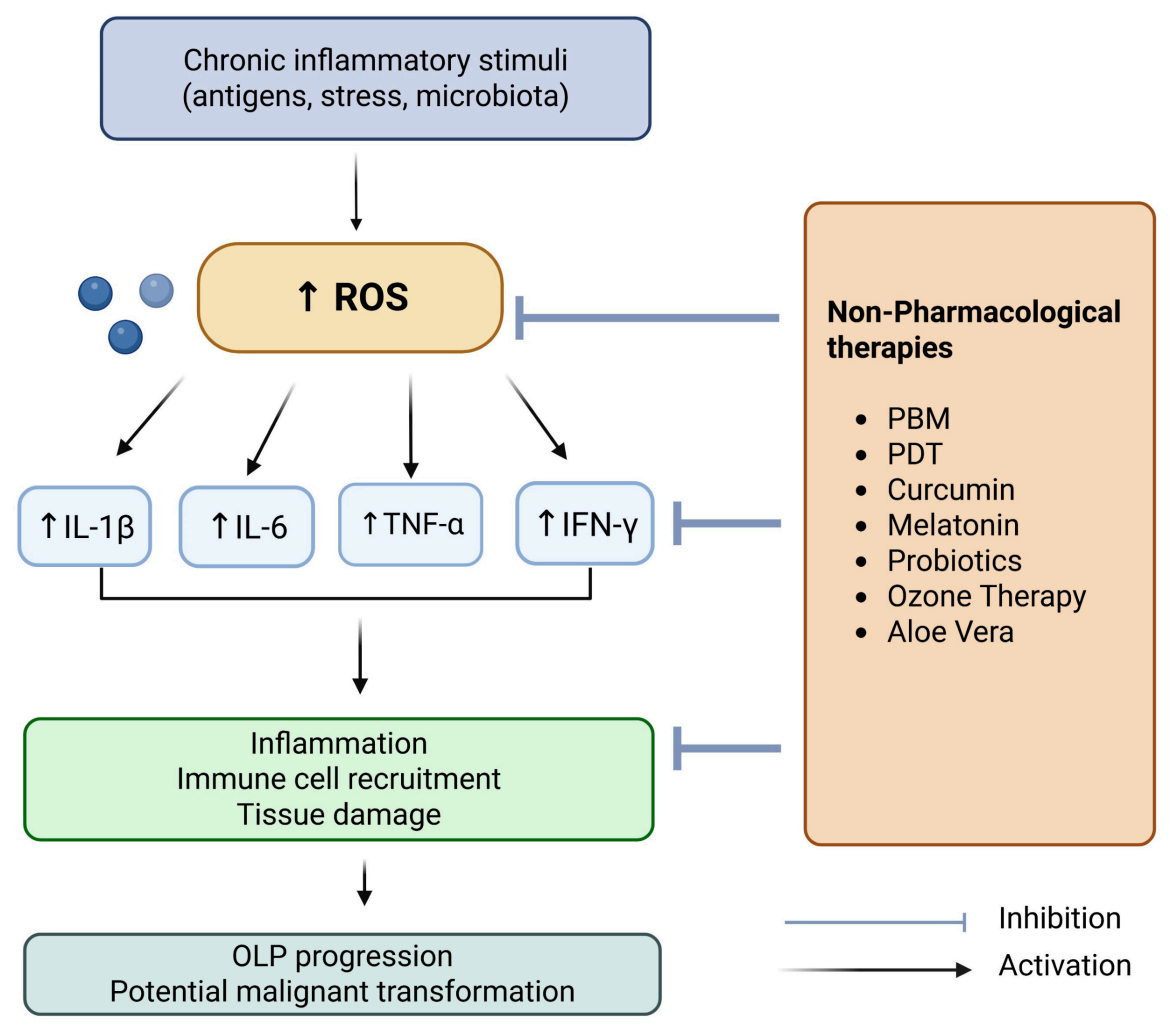
Pro-inflammatory cytokines and reactive species oxygen (ROS) in Oral lichen planus (OLP): sources and therapeutic modulation. Created with https://BioRender.com/2bjp36w on 29 July 2026, Re, F. (2026). PBM: photobiomodulation; PDT: photodynamic therapy; interleukin-1β: IL-1β; interleukin-6: IL-6; tumor necrosis factor-α: TNF-α; interferon-γ: IFN-γ.

Taken together, this body of evidence positions PBM as a clinically validated, well-tolerated alternative or adjunct to topical corticosteroids in OLP management, particularly well-suited to steroid-resistant or steroid-intolerant patients. Persistent heterogeneity in wavelength, power density, energy fluence, and treatment frequency across studies remains the principal obstacle to defining a single optimized protocol and represents the most pressing methodological gap for future dose-finding trials to address [33]. Figure 2 summarizes the main cytokine- and reactive oxygen species (ROS)-related pathways targeted by the non-pharmacological interventions discussed in this review.

### 3.3. Photodynamic Therapy

PDT employs a light-activated photosensitizer to generate reactive oxygen species that selectively induce apoptosis of inflammatory cells within the lesion. A systematic review found that PDT reduced the lesion size by a mean of 1.53 cm^2^, achieved a partial response rate of 0.77, and decreased the visual analog scale (VAS) pain score by 3.82 points and the Thongprasom sign score by 1.33 points; subgroup analyses indicated that 5-aminolevulinic acid (5-ALA) achieved a better response than methylene blue as a photosensitizer, and that PDT was overall as effective as topical corticosteroid therapy [34].

A three-arm randomized clinical trial comparing PDT, PBM, and triamcinolone acetonide 0.1% found that improvement in oral health-related quality of life was sustained at 3 months in both the PDT and PBM groups, whereas symptoms returned in the corticosteroid group over the same follow-up period [35]. A prospective case–control pilot study further demonstrated that PDT produced significant reductions in lesion size, ABSIS score, and Thongprasom score alongside improvements in quality of life, and that these changes correlated with a significant decrease in lesional CD4+ and CD8+ T-cell infiltration, supporting an immunomodulatory mechanism of action [36].

The reported adverse effects of PDT are generally mild and transient, including localized pain, swelling, and erythema, typically decreasing across subsequent treatment sessions. The principal limitations of this modality are the requirement for specialized equipment, the associated cost, and the relatively small sample sizes of available studies, which constrain the generalizability of efficacy estimates [36].

Table 1 outlines the available light-based treatments for OLP, detailing their mechanisms of action, clinical uses, and evidence levels.

## 4. Nutraceutical and Phytotherapeutic Agents

Nutraceutical and phytotherapeutic agents have been investigated in OLP primarily for their antioxidant, anti-inflammatory, and immunomodulatory properties, based on the rationale that compounds capable of modulating the same cytokine and oxidative-stress pathways implicated in OLP pathogenesis might offer a better-tolerated alternative or adjunct to corticosteroid therapy. This category encompasses an unusually wide range of agents and levels of evidentiary maturity, spanning the comparatively extensive, though markedly inconsistent, curcumin literature to single mechanistic or pilot-level studies for emerging candidates such as melatonin and flaxseed extracts.

### 4.1. Curcumin

Curcumin (diferuloylmethane), the principal polyphenolic pigment of *Curcuma longa*, is among the most extensively investigated phytotherapeutic agents for OLP [37,38]. Its anti-inflammatory activity is mediated through downregulation of the NF-κB signaling pathway, inhibition of cyclooxygenase-2 (COX-2) and 5-lipoxygenase, and suppression of pro-inflammatory cytokines including TNF-α, IL-1β, IL-6, and interleukin-8 (IL-8) [37,38]. In parallel, curcumin exerts antioxidant effects through direct scavenging of reactive oxygen and nitrogen species and activation of the nuclear factor erythroid 2-related factor 2 (Nrf2) pathway, resulting in upregulation of endogenous antioxidant enzymes such as superoxide dismutase and glutathione peroxidase [37,38]. These mechanisms are particularly relevant given the recognized role of oxidative stress in OLP pathogenesis and malignant transformation. Additional antimicrobial and antifungal properties have also been proposed to have potential advantages over corticosteroid therapy, which may predispose patients to secondary oral candidiasis. The main obstacle to clinical translation is poor oral bioavailability, as curcumin is poorly soluble, unstable at physiological pH, and rapidly metabolized on first pass. As shown below, formulation-dependent bioavailability, rather than nominal dose, appears to be the strongest predictor of clinical efficacy [38].

Systemic curcuminoids show a clear dose-dependent pattern. At 2000 mg/day, two independent placebo-controlled trials found no benefit: one was stopped early for futility [39], and the other used concurrent corticosteroids in both arms, which may have masked any added effect [40]. At 6000 mg/day without background steroid, the same research group found significant improvement in signs and symptoms [41]. After long-term follow-up, roughly 60% of patients reported sustained benefit, with adverse effects limited mainly to mild, dose-related gastrointestinal symptoms [42].

Topical curcumin formulations generally match the efficacy of, but do not clearly outperform, topical corticosteroids. A 5% curcumin paste performed comparably to 0.1% triamcinolone acetonide [43], and a curcumin mucoadhesive paste matched the performance of betamethasone plus nystatin [44]. Efficacy also appears frequency-dependent; for example, a 1% curcumin gel applied six times daily nearly achieved corticosteroid-induced levels of improvement, while the same gel applied only three times daily underperformed [45]. An uncontrolled pilot study reported benefit from topical turmeric ointment [46]. None of these topical trials were blinded, which complicates interpretation given OLP’s tendency toward spontaneous remission. Nanoformulated curcumin, designed to overcome the bioavailability problem, shows an encouraging early signal. Oral nanocurcumin at just 80 mg/day achieved improvement comparable to prednisolone 10 mg/day [47]; this dose is roughly 25-fold lower than the ineffective conventional doses mentioned above, which is consistent with bioavailability driving efficacy. Adding 1% nanocurcumin gel to a triamcinolone rinse produced numerically greater, though not always statistically significant, improvement compared with a placebo gel plus the same rinse [48]. Compared with these positive individual trials, systematic appraisals are more cautious. A meta-analysis of 10 studies and 355 patients found no significant overall benefit regarding pain, erythema, or lesion size, with the largest apparent effects concentrated in non-randomized and short-duration trials [49], suggesting that some reported benefits reflect methodological weakness rather than true biological effects. Curcumin is otherwise well tolerated even at high doses [37,50]. Overall, curcumin remains biologically plausible but evidentially unproven, with nanoformulation the most promising route to resolving the gap between positive individual trials and null pooled estimates [49].

### 4.2. Aloe Vera

Aloe vera (*Aloe barbadensis* Miller) is among the most extensively investigated phytotherapeutic agents for OLP [51]. Its proposed therapeutic effects include anti-inflammatory, analgesic, immunomodulatory, and wound-healing activities mediated by compounds such as acemannan, aloe-emodin, C-glucosyl chromone, and other polysaccharides, which modulate inflammatory pathways and promote tissue repair [52]. Despite this strong biological rationale, clinical outcomes have been inconsistent.

The most recent randomized controlled trial compared topical and systemic aloe vera with topical clobetasol propionate in 60 patients with OLP [52]. Both groups showed significant clinical improvement, although clobetasol produced greater short-term reductions in pain and disease severity [52]. Interestingly, symptom recurrence during follow-up appeared more pronounced in the corticosteroid group, whereas improvements were largely maintained in the aloe vera group. No adverse effects were reported with aloe vera [52]. However, the absence of a placebo arm limits the interpretation of efficacy.

A separate three-arm study compared the effects of aloe vera with turmeric and *Withania somnifera* on symptomatic gingival OLP. All treatments significantly reduced pain and lesion size, with no significant differences between groups [53].

The most rigorous placebo-controlled trial evaluated a 70% aloe vera suspension versus placebo for 12 weeks [54]. Aloe vera failed to demonstrate significant superiority for pain reduction, clinical severity, or overall treatment response. Nevertheless, improvements in oral health-related quality of life, particularly psychological domains, were observed, while the marked response in the placebo arm highlighted the importance of placebo effects in OLP [54].

The broader evidence base was summarized in a systematic review and meta-analysis including seven studies and 196 patients [55]. The earliest placebo-controlled trial reported significantly greater pain remission and clinical response with aloe vera than with placebo [56]. In contrast, studies comparing aloe vera with triamcinolone produced conflicting results, with one reporting superiority of aloe vera [57,58] and another finding no significant differences between treatments [59]. Meta-analytic findings were similarly inconsistent, reflecting the small number of studies and substantial methodological heterogeneity [55].

More recently, aloe vera was compared with low-level laser therapy [59]. Laser therapy produced greater improvement during active treatment, but the differences disappeared during long-term follow-up, as the benefits in the aloe vera group continued after treatment cessation [59].

Overall, the evidence supporting aloe vera in OLP remains heterogeneous. Randomized studies have reported aloe vera to be superior [57], comparable [58], or inferior [52] to corticosteroid therapy, while placebo-controlled trials have yielded conflicting findings [54,56]. Interpretation is further complicated by marked variation in formulations and treatment protocols across studies. Importantly, the most rigorously blinded trial failed to demonstrate superiority over placebo regarding its primary clinical outcomes [54]. Nevertheless, aloe vera has consistently shown an excellent safety profile, with no significant adverse events reported across available studies [52,54]. It may therefore be considered a safe adjunctive option for OLP, although its efficacy relative to placebo and established therapies remains uncertain.

### 4.3. Coconut (Cocos nucifera)

Coconut (*Cocos nucifera*) has been proposed as a topical alternative to corticosteroids in OLP because of its anti-inflammatory, antioxidant, immunomodulatory, and wound-healing properties. These effects have been attributed to its medium-chain fatty acids, particularly lauric, palmitic, and capric acids, as well as a mannose-6-phosphate-rich glycoprotein and polysaccharide fraction involved in tissue repair [60]. However, unlike curcumin and melatonin, evidence supporting coconut use in OLP remains largely extrapolated from dermatological and wound-healing research, with no OLP-specific mechanistic studies currently available.

The first randomized double-blind trial compared a 50% *w*/*v* coconut cream with 0.05% clobetasol propionate in 60 patients over 2 months [60], where both treatments significantly reduced lesion size and pain [60]. Although the coconut group showed greater early improvement, baseline lesion severity was substantially higher, complicating interpretation of comparative efficacy. By 60 days, both treatments achieved marked clinical improvement, with clobetasol showing numerically greater lesion regression (95% vs. 85%), while coconut cream achieved complete pain resolution and no adverse effects were reported [60].

A subsequent three-arm randomized trial compared the same 50% coconut cream with pomegranate peel-extract gel and 0.1% tacrolimus ointment in 30 patients with symptomatic gingival OLP [61]. All groups demonstrated significant reductions in pain and lesion size, with no significant differences between treatments. Improvement in burning sensation was observed within 2 weeks of coconut therapy, although refrigeration and limited shelf life were identified as practical limitations [61].

More recently, a coconut–licorice–purslane (CLP) cream was compared with 0.1% triamcinolone acetonide gel in 40 randomized patients [62]. Both groups showed significant pain reduction. However, baseline pain severity was lower in the CLP group, and when improvement from baseline was analyzed, triamcinolone produced significantly greater benefit at all major follow-up points [62]. Consequently, the study does not clearly support the superiority of the CLP formulation, despite the authors’ conclusions [62].

Overall, evidence supporting coconut-based therapies in OLP remains limited. No placebo-controlled studies are available, and baseline imbalances favoring coconut-based formulations were observed in the principal comparative trials [60,62]. Nevertheless, studies have consistently used a similar 50% cream formulation, allowing greater comparability across investigations. Given the absence of OLP-specific mechanistic evidence and the methodological limitations of existing trials, coconut cream should currently be regarded as a promising but preliminary adjunctive option rather than an established non-pharmacological treatment for OLP.

### 4.4. Melatonin

Melatonin (N-acetyl-5-methoxytryptamine, MLT), an indoleamine produced by the pineal gland, exerts antioxidant, anti-inflammatory, and immunomodulatory effects through both receptor-dependent and receptor-independent pathways and has been implicated in the NF-κB signaling network involved in OLP pathogenesis [63]. In addition to its pineal origin, MLT can be synthesized locally by extra-pineal tissues, such as the skin (keratinocytes), oral/gastrointestinal mucosa, salivary glands, bone marrow, retina, and activated immune cells, suggesting a potential tissue-level protective role within inflammatory lesions [64,65].

Evidence for altered MLT biology in OLP comes from an immunohistochemical case—control study comparing 30 OLP biopsies with 30 normal oral mucosal specimens [63]. Expression levels of MLT, as assessed by immunohistochemical staining with a semiquantitative scoring system, its synthesizing enzyme arylalkylamine-N-acetyltransferase (AANAT), and its receptor MT1 were significantly higher in OLP tissues than in controls. Moreover, expression extended beyond the epithelium into the inflammatory infiltrate, supporting the concept of local MLT production within OLP lesions [63]. No significant differences in immunostaining scores for AANAT, melatonin, and MT1 were observed between atrophic and erosive lesions or between sexes.

Building on these findings, a subsequent hypothesis paper proposed an “immune-pineal axis” model [66], in which NF-κB-driven inflammatory signaling shifts the source of melatonin from pinealocytes toward locally activated oral epithelial and immune cells. According to this framework, locally produced MLT may have compensatory antioxidant and cytoprotective effects within the chronic inflammatory microenvironment of OLP, although this mechanism remains speculative and has not been experimentally validated.

In contrast, a pilot case–control study reported significantly lower salivary MLT levels in OLP patients than in healthy controls, with the lowest concentrations in atrophic/erosive disease [67]. Because salivary flow was also reduced, this may partly reflect impaired salivary gland function rather than reduced MLT production per se.

According to this model, locally produced MLT may exert compensatory antioxidant and cytoprotective effects within the chronic inflammatory microenvironment of OLP. However, this mechanism remains hypothetical and has not yet been experimentally validated [63].

Taken together, the available evidence indicates that MLT biology is altered in OLP, but the direction of this alteration is compartment-dependent; that is, it differs according to whether melatonin is measured in lesional tissue or in saliva. In inflamed mucosal tissue, immunohistochemistry shows increased expression of MLT, its synthesizing enzyme AANAT, and the MT1 receptor [63], whereas salivary MLT levels appear reduced, particularly in atrophic/erosive disease [67]. These opposite trends are not necessarily contradictory; according to the immune–pineal axis hypothesis (Markus et al., 2013) [66], inflammatory signaling (TNF-α, NF-κB) suppresses systemic, pineal-derived melatonin levels while simultaneously driving its local synthesis by activated oral epithelial and immune cells. Chaiyarit et al. [64] adapted this model to OLP, proposing that reduced circulating/salivary melatonin and increased lesional melatonin may represent two facets of a single compensatory shift from systemic to local production, in which locally generated MLT exerts antioxidant and cytoprotective effects within the chronic inflammatory microenvironment. Whether the divergent findings truly reflect this regulatory shift or are partly driven by methodological limitations, for example, the reduced salivary flow that accompanies severe OLP, cannot yet be determined. Nevertheless, the consistent association between melatonin dysregulation and OLP has generated interest in its therapeutic potential: a registered randomized clinical trial is currently evaluating oral MLT supplementation as an adjunct to conventional therapy, assessing both clinical outcomes and DNA hypermethylation as a potential marker of malignant transformation risk [64].

### 4.5. Zinc and Vitamin D Supplementation

Among micronutrient-based approaches, zinc and vitamin D have received the most direct clinical investigation. A three-arm randomized clinical trial with 42 patients compared topical triamcinolone acetonide alone with triamcinolone plus systemic zinc sulfate (50 mg/day) or triamcinolone plus systemic vitamin D (2800 IU/week) over 8 weeks. By week 3, pain scores were significantly lower in both the zinc and vitamin D groups than in the corticosteroid-only group, and by week 8 the zinc group showed the most pronounced reduction in Thongprasom score (median 0) compared with vitamin D (median 1) and corticosteroid alone (median 1.5); no adverse effects were observed in any group [68]. These findings are broadly consistent with an earlier trial of vitamin D supplementation in peri-menopausal women with OLP, which reported significant pain reduction in both treatment arms but significant clinical improvement only with adjunctive vitamin D [69], and with a double-blind randomized trial in which vitamin D supplementation, as an adjuvant to conventional therapy, produced more pronounced and earlier pain reduction and significant clinical improvement compared with placebo [70].

### 4.6. Other Topical Nutraceutical Formulations

A topical mouthwash containing hyaluronic acid, calcium hydroxide, umbelliferone, and oligomeric proanthocyanidins has also been compared directly with clobetasol gel in a randomized trial [71]. Overall, nutraceutical formulations are characterized by biologically plausible mechanisms and an expanding body of clinical trial evidence, particularly for curcumin, aloe vera, zinc, and vitamin D, but considerable heterogeneity in formulation, dosing, and outcome measures continues to limit firm efficacy conclusions, and several agents remain at an earlier, primarily mechanistic or preclinical stage of investigation. Due to the significant differences in their biological targets and clinical support, Table 2 offers a comparative overview of the primary characteristics of nutraceutical interventions.

## 5. Cryotherapy and Ozone Therapy

Cryotherapy induces controlled, non-surgical tissue destruction through rapid freezing, avoiding the procedural pain associated with scalpel or laser excision, while ozone therapy is proposed to act through immunomodulatory, antioxidant, antimicrobial, and wound-healing-promoting mechanisms, partly mediated via activation of the Nrf2 antioxidant pathway. A randomized trial comparing cryotherapy using nitrous oxide gas with topical corticosteroids in patients with bilateral lesions found comparable improvements in appearance score, VAS pain, and lesion severity between the two approaches [72]. In a four-arm comparative study of surgical modalities for oral potentially malignant disorders, cryotherapy was associated with lower postoperative pain than diode laser, CO2 laser, or scalpel surgery both on the day of treatment and after 1 week of follow-up [73]. A direct comparison between LLLT and ozone therapy further situates ozone within the broader landscape of light- and gas-based non-pharmacological approaches, providing one of the few head-to-head comparisons available between treatment categories considered in this review [74].

As a strong oxidizing agent, ozone has been proposed as a treatment for OLP because of its antimicrobial, anti-inflammatory, and tissue-repair properties. At therapeutic concentrations, it is also reported to stimulate protective cellular pathways and cytokine production, potentially contributing to tissue regeneration and modulation of the dysregulated immune response that is characteristic of OLP [75]. Because gaseous ozone may cause inhalation toxicity and uncontrolled dispersion, clinical applications generally employ ozonized water or ozonized oil to localize the therapeutic effect [75].

Clinical evidence remains limited to four randomized trials and one longitudinal case series identified in a 2024 systematic review [75]. In the largest randomized trial, ozonized water used as an adjunct to topical betamethasone resulted in greater clinical improvement and lower relapse rates than placebo, although both groups improved during follow-up [76]. A three-arm trial comparing ozone, triamcinolone acetonide, and combined ozone-plus-triamcinolone therapy found significant improvement with corticosteroid treatment alone and, most notably, with combination therapy, whereas ozone monotherapy did not achieve significant benefit, suggesting an adjunctive rather than an independent therapeutic effect [77].

Two split-mouth studies in bilateral reticular OLP reported substantial clinical improvement with ozone therapy. In one trial, both ozone- and corticosteroid-treated lesions achieved complete resolution, with somewhat greater erythema reduction on the ozone-treated side [78]. A second study similarly reported complete lesion resolution and improvement in pain scores following ozone therapy, although inconsistencies regarding the comparator treatment limit interpretation of the findings [74]. A longitudinal case series using ozonized olive oil reported symptomatic improvement, but only five OLP patients were included, and no control group was available [79].

Overall, the evidence supporting ozone in OLP remains limited. The only dedicated systematic review identified a high risk of performance bias across all randomized trials because participant and personnel blinding was not feasible [79]. Moreover, the only study specifically evaluating ozone monotherapy failed to demonstrate significant efficacy, whereas combination therapy with corticosteroids produced clear clinical benefit [80]. Additional limitations include the small number of studies, heterogeneity in ozone formulations and delivery protocols, and reliance on split-mouth designs in predominantly reticular disease [74,78,79]. Consequently, ozone should currently be regarded as a promising but preliminary adjunctive therapy, with the available evidence supporting its use alongside rather than instead of topical corticosteroids [75,79].

## 6. Probiotics and Oral Microbiome-Targeted Approaches

Interest in probiotic and oral microbiome-targeted approaches stems from accumulating evidence linking oral microbial dysbiosis to OLP pathogenesis and malignant transformation potential, which has been estimated to affect between 0.44% and 2.8% of patients with OLP [81]. A comprehensive non-systematic review of Lactobacilli and Bifidobacteria strains and their metabolites described multiple convergent mechanisms by which probiotics may modulate OLP-relevant pathways, including suppression of excessive effector T-cell responses and restoration of the Treg/Th17 balance, reduction in NF-κB-dependent pro-inflammatory cytokine signaling (including IL-6, IL-8, and TNF-α), downregulation of matrix metalloproteinase-9 (a protease implicated in basal membrane breakdown and disease progression), and attenuation of basal keratinocyte apoptosis [81]. An earlier review specifically framed probiotics as a “non-conventional therapy” for OLP, noting their ability to modulate microbial infection predisposition, T-cell activation and infiltration, and mast cell degranulation in a strain-specific manner [82]. At the cellular level, exposure of an oral squamous carcinoma cell line to *Streptococcus salivarius* supernatant significantly reduced IL-6 expression and showed a decreasing trend for IL-1β, IL-8, and TNF-α expression levels, consistent with inhibition of the NF-κB pathway, a mechanistic finding that directly informed the clinical observations described below [83].

Direct clinical evidence for probiotic use in OLP remains limited but is accumulating. An observational and controlled clinical study found that *Streptococcus salivarius* was relatively depleted in the oral microbiota of patients with OLP compared with healthy controls, and that supplementation with *S. salivarius* K12 reduced symptoms in the OLP cohort studied [83]. In a separate randomized clinical trial, supplementary oral probiotic capsules added to topical clobetasol propionate 0.05%, compared with clobetasol alone, produced a significantly greater reduction in both numerical pain rating scale score and Thongprasom score, with the benefit already apparent at 2 weeks and more pronounced at 4 weeks [84]. A 1-year longitudinal randomized trial investigating the oral bacteriome and mycobiome in OLP patients receiving conventional treatment with or without probiotic supplementation found considerable inter-individual variability in microbial composition over time, with the lesional cytobrush-derived microbiome appearing less influenced by treatment than the whole-mouthwash-derived sample [85].

This treatment approach is now supported by a small but growing number of direct clinical trials, in addition to a substantial mechanistic and preclinical body of literature; however, sample sizes remain modest, follow-up periods short, and protocols (strain selection, dose, and duration) highly heterogeneous. Larger and longer-duration clinical trials are needed to confirm whether microbiome-targeted interventions can meaningfully alter disease course, including recurrence and malignant transformation risk.

## 7. Psychological and Behavioral Interventions

A well-documented bidirectional relationship exists between psychological distress and OLP, with chronic stress, anxiety, and depression implicated both as potential risk factors for disease onset/exacerbation, plausibly via hypothalamic–pituitary–adrenal axis activation and downstream immune effects, and as consequences of living with a chronic, visible, and painful oral condition [86].

In terms of direct intervention evidence, a randomized trial evaluating stress-control techniques, Jacobson’s Progressive Muscle Relaxation, or an herbal sedative, in addition to standard pharmacological treatment, in patients with high perceived stress found a significant reduction in both perceived stress (Perceived Stress Scale) and pain compared with patients without high stress levels who received standard treatment alone [87]. Supporting observational evidence comes from a case series of six patients for whom psychological counseling combined with corticosteroid therapy was associated with clinical improvement of OLP lesions alongside reductions in depression, anxiety, and stress scores measured using the DASS-21 instrument [88], and from a case report in which a combination of psychotropic medication and psychotherapy resolved a corticosteroid-refractory lesion in a patient with underlying major depressive disorder, illustrating a potential treatment pathway for cases unresponsive to conventional oral-directed therapy alone [89].

This category remains the least supported by controlled intervention trials among those considered in this review, with most available evidence derived from observational or case-level designs; nonetheless, the consistency of the association between psychological distress and OLP severity provides a strong rationale for incorporating psychological screening and, where indicated, targeted intervention into comprehensive OLP management.

Table 3 provides a comparative summary of adjunctive non-pharmacological methods.

## 8. OSCC Diagnosis and Follow-Up

The diagnosis of OSCC consists of medical history, clinical examination, histological examination, and imaging studies to investigate possible locoregional and/or distant spread [90].

Several aspects should be considered in the medical history, including age, tobacco use (smoking or chewing), alcohol consumption, chronic trauma, poor oral hygiene, and a family or personal history of neoplasms in the same or different sites. The physical examination is based on inspection and thorough palpation of the entire oral mucosa, including the following sites: lips (vermilion border), tongue, cheeks, floor of the mouth, retromolar triangle, hard and soft palate, and gingival mucosa. In addition to the oral cavity, palpation should be extended to the cervical lymph nodes of the neck, paying attention to the presence of masses that may represent lymph node metastases [18,91].

OSCC lesions vary in size, ranging from a few millimeters to several centimeters in more advanced cases. Malignant lesions in the early stages may present as leukoplastic, erythroplastic, or erythro-leukoplastic lesions, consisting of red, white, or red and white areas with a slight roughness. The elasticity of the soft tissues is reduced to the point that a sensation of “hardening” can be felt on palpation. Often the lesion is not painful, but it may be tender [91].

At advanced stages, oral neoplasms often present with characteristics such as ulceration, nodularity, and fixation to underlying tissues; they may take the form of a mass with a verrucous surface and poorly defined borders. In these cases, they are often associated with lymph node metastases in the neck region. Furthermore, the lesion is usually painful and/or tender to palpation. At the conclusion of the physical examination, if a lesion of suspected malignancy is present, a biopsy is scheduled for histological analysis of the tissue. The imaging tests performed are computed tomography (CT) and magnetic resonance imaging (MRI). CT and MRI scans of the facial skeleton and neck, both with and without contrast, are used to determine the extent of the primary tumor; they also evaluate the lateral cervical lymph nodes. Ultrasound-guided fine needle aspiration, indicated for the evaluation of lymph node involvement in cases of tumors of unknown origin, is performed of the lateral cervical lymph nodes affected by the disease and subsequently sent for cytological examination [92].

For the evaluation of distant secondary lesions, the following are indicated: abdominal ultrasound and chest X-ray in two projections as first-level tests in the evaluation of initial diseases (Stages I and II); and Level II tests, such as whole-body CT, abdominal MRI, and positron emission tomography (PET)/CT, which are indicated for the evaluation of advanced disease (Stages III and IV) [93].

## 9. Non-Pharmacological Treatment of OSCC

Non-pharmacological treatments for OSCC include mostly surgery and, if required, postoperative radiation. For oral carcinomas, surgery is typically the first line of treatment. Postoperative radiation may be used in advanced cases [94]. The goal of treatment is two-fold; namely, to ensure oncological control of the disease and to preserve, as much as possible, the vital functions of the oral cavity, such as speaking, swallowing, and chewing.

### 9.1. Surgical Techniques

A large percentage of oral carcinomas are treated non-pharmacologically with surgical techniques such as robotic surgery, endoscopic procedures, and open procedures. The aim of surgical resection is to remove enough tumor tissue. Inadequate tumor cell removal decreases long-term survival rates by increasing the risk of local and regional recurrences [94,95].

After removing the primary tumor, reconstructive surgery is often necessary to restore the function of the oral cavity, as well as the appearance of the head and neck. Regular surgical reconstruction can help mitigate postoperative oral disabilities. The selection of a suitable reconstruction method is influenced by several factors, including the characteristics of the primary defects, the patient’s medical history and overall health, the surgeon’s expertise, and the expected outcome [96].

The aim of surgical treatments is to achieve complete tumor excision with histologically negative margins (R0), which is the main prognostic factor for local control and long-term survival. Depending on tumor location and extent, surgical procedures include partial or total glossectomy, marginal or segmental mandibulectomy in cases of bone invasion, and composite resections involving adjacent soft tissues and bone. Positive surgical margins (R1) are associated with an increased risk of local recurrence and poorer overall survival [97,98,99].

Reconstructive surgery is an integral component of OSCC treatment, particularly after extensive resections of the oral cavity and oropharynx.

Typically, reconstructive procedures follow a “reconstructive ladder,” which starts with a skin graft and progresses to a microvascular free flap. In oral reconstruction, unrestricted tissue transfer is one of the most dependable and commonly utilized techniques [100]. Microvascular free flaps, such as the fibula, radial forearm, and anterolateral thigh flaps, represent the current standard of care for restoring both hard and soft tissue defects. Beyond aesthetic reconstruction, these techniques significantly improve speech, swallowing, oral function, and quality of life, facilitating earlier rehabilitation and social reintegration. Optimal outcomes require multidisciplinary planning involving head and neck surgeons, reconstructive microsurgeons, speech therapists, nutrition specialists, and rehabilitation teams [101].

A 1 cm margin for a three-dimensional dissection is considered adequate in oral cancer surgery [102].

Iodine solution staining is advised for the identification and delineation of the dysplastic epithelium during primary tumor dissection [103]. In cases of OSCC, obtaining sufficient surgical margins frequently necessitates larger resections, potentially leading to higher postoperative complications. When appropriate, adjuvant therapy should commence within 6 to 7 weeks post-surgery to enhance oncological results [104]. A longer timeframe may be justified in cases of slow-healing surgical complications (e.g., wound dehiscence, fistula formation); however, postoperative radiation therapy should be administered as soon as possible to decrease the risk of functional and aesthetic-related side effects [105].

### 9.2. Radiotherapy

Radiotherapy is an integral component of OSCC management and, alongside surgery, represents one of the two primary non-pharmacological treatment modalities for this disease. It may be employed as definitive, adjuvant (postoperative), or palliative treatment, depending on tumor stage, resectability, and histopathological risk features [106]. Because surgery remains the standard of care for resectable disease, radiotherapy is most commonly delivered postoperatively; primary radiotherapy, with or without concurrent chemotherapy, is reserved for unresectable tumors or for patients who are medically unfit for surgery. The strongest established indications for postoperative radiotherapy are the presence of positive or close surgical margins and extranodal extension of nodal disease, both of which are independently associated with increased locoregional recurrence risk [107]. Additional adverse features, including advanced tumor stage (pT3–pT4), depth of invasion greater than 4 mm, perineural invasion, and lymphovascular invasion, may individually or in combination also support the use of adjuvant treatment. From a technical standpoint, modern conformal delivery methods, particularly intensity-modulated radiotherapy (IMRT) and volumetric modulated arc therapy (VMAT), allow concentration of the prescribed dose on the target volume while limiting exposure to critical structures such as the salivary glands, spinal cord, and oropharyngeal mucosa, thereby reducing late complications including xerostomia, dysphagia, and osteoradionecrosis without compromising oncological outcomes [106,107,108]. Timely initiation is critical; in particular, postoperative radiotherapy should ideally begin within 5 to 7 weeks of surgery, as delays in the overall treatment package time are independently associated with worse locoregional control.

### 9.3. Photobiomodulation

PBM refers to the application of low-energy red or near-infrared light (roughly 600–950 nm, the so-called “optical window”) to stimulate physiological repair processes: photons are absorbed by mitochondrial cytochrome c-oxidase, leading to increased ATP production, moderate generation of ROS, and release of nitric oxide [23]. This mechanism has made PBM a widely used adjunct in dentistry for managing oral mucositis and other side effects of chemo- and radiotherapy; however, its use in patients with oral squamous cell carcinoma (OSCC) remains controversial as the same proliferative stimulus that promotes healing of healthy tissue could, in principle, also encourage the growth of neoplastic cells [23]. A systematic review by Del Vecchio et al. [23] based on 12 in vitro and in vivo studies reports genuinely conflicting evidence—some authors describe PBM as stimulating tumor growth or increasing the aggressiveness of dysplastic and neoplastic cell lines, while others report beneficial effects, including direct cytotoxic action toward cancer cells at high fluences (exploiting the biphasic dose–response curve, or “Arndt–Schulz law,” whereby low doses stimulate while higher doses inhibit or trigger apoptosis), a differential effect between healthy and neoplastic cells linked to the Warburg effect, and possible stimulation of the immune system via modulation of pro-inflammatory mediators. The authors conclude that, while risks cannot be entirely excluded, there is real potential for PBM to play a beneficial role in cancer patient management, provided correct protocols and dosages are identified [23]. More recent studies tend to reinforce this cautiously favorable picture. An in vivo study using a 4-nitroquinoline-N-oxide (4NQO)-induced rat model of oral carcinogenesis tested PBM protocols commonly used for mucositis management (0.3 J and 1 J, 660 nm) and found no significant differences compared with controls in terms of lesion progression, histopathological features, or expression of p53 and ROS1, suggesting that these standard mucositis protocols do not accelerate oral carcinogenesis [109]. On the in vitro side, a study of Cal-27 OSCC cells exposed to a 660 nm diode laser showed that specific parameters (20 mW, 2 J/cm^2^, 30 s) significantly reduced cell viability and increased apoptosis relative to controls, whereas higher power and shorter exposure times (at the same energy density) did not produce a comparable effect, confirming that outcomes depend strongly on the specific physical parameters used (wavelength, power, fluence, exposure time), not merely on total delivered energy [110]. A further line of research explores PBM not as a standalone treatment, but as a synergistic pretreatment for other therapies: in OSCC cell cultures, PBM pretreatment (635 or 830 nm) produced a dose-dependent increase in 5-aminolevulinic acid-induced protoporphyrin IX accumulation and significantly enhanced the cytotoxic response to subsequent photodynamic therapy (PDT), without inducing meaningful toxicity on its own, pointing to a mechanism based on stimulation of mitochondrial activity rather than a simple additive light effect [111]. Taken together, the evidence from the current literature indicates that PBM’s effects on oral carcinoma are strongly dose- and parameter-dependent. While there is not yet definitive, generalizable clinical proof of safety, several potentially advantageous applications are emerging, whether as a tool for managing treatment side effects without accelerating disease progression, as a selective cytotoxic agent under high-fluence regimens, or as a sensitizer that potentiates other photoactivated therapies. Standardization of protocols (wavelength, power, fluence, delivery mode) and further controlled clinical trials remain necessary before firm conclusions can be drawn about the clinical use of PBM in patients with OSCC.

### 9.4. Oral Hygiene Management

For patients with OSCC, maintaining good oral hygiene is an essential part of supportive care, as it promotes treatment continuity and increases the probability of completing oncological regimens [112].

Dental professionals are crucial in preventing and treating oral problems such as dental caries, periodontal disease, oral candidiasis, mucositis, salivary dysfunction, osteoradionecrosis, dysphagia, and mouth pain across the whole treatment pathway, from diagnosis to survivorship [113].

Despite the availability of standardized oral assessment instruments, there is currently no widely recognized oral hygiene protocol, and routine clinical adoption of such protocols is still limited [113]. In order to reduce the risk of treatment-related issues, pre-treatment dental evaluation is highly advised in order to detect and treat periodontal disease and mouth infections prior to surgery or radiation [114].

The incidence of radiation-induced dental caries linked to xerostomia after irradiation has been demonstrated to be reduced by preventive treatments, such as topical fluoride administration. Similarly, the Multinational Association Supportive Care Cancer/International Society Oral Oncology (MASCC/ISOO) guidelines encourage systematic oral hygiene procedures as part of supportive cancer care, since they reduce the severity of oral mucositis [115]. By minimizing the oral microbial burden, maintaining good oral hygiene also helps to reduce the incidence of surgery site infections [116]. Additionally, to reduce the incidence of osteoradionecrosis, one of the most serious long-term side effects of head and neck irradiation, good dental management is crucial both before and after radiotherapy [117]. Another important component of managing oral health is patient education. Oral health education, treatment adherence, quality of life, and long-term functional results in OSCC survivors are all improved by self-care, caregiver involvement, and multidisciplinary teamwork among dentists, nurses, physicians, and allied medical personnel [118].

### 9.5. Post-Treatment Rehabilitation

In order to improve overall quality of life, promote social reintegration, and restore oral function, post-treatment rehabilitation is a crucial part of the multidisciplinary management of patients with OSCC. Early rehabilitation is essential across the continuum of care since surgical resection and radiotherapy may result in speech impairment, dysphagia, trismus, nutritional deficits, and psychological discomfort. It has been demonstrated that a multidisciplinary strategy incorporating maxillofacial prosthodontists, speech therapists, physiotherapists, nutritionists, and psychologists improves functional outcomes, decreases long-term problems, and increases treatment adherence [119].

Speech and swallowing rehabilitation greatly enhances communication and swallowing function while decreasing the risk of aspiration. This includes tongue mobility exercises, respiratory–phonatory training, compensatory swallowing techniques, and instrumental assessment with videofluoroscopy or fiberoptic endoscopic evaluation of swallowing (FEES) [119]. Since malnutrition affects over half of patients with OSCC and adversely impacts treatment tolerance and survival, nutritional screening and early personalized nutritional support are equally critical. In order to prevent or treat trismus after surgery or radiation therapy, physical rehabilitation, especially jaw-opening exercises and physiotherapy, is essential [120].

Additionally, mastication, speech, facial aesthetics, and psychological well-being are all restored by maxillofacial prosthetic rehabilitation, which is increasingly supported by CAD/CAM technology and three-dimensional printing [121,122]. Finally, as anxiety, depression, and body image issues are prevalent among OSCC survivors and may have a detrimental impact on long-term quality of life and adherence to follow-up, psychological support should be incorporated into rehabilitation programs [123,124]. By reducing complications, hospital readmissions, and long-term disability, early interdisciplinary rehabilitation not only enhances functional recovery, but may also reduce healthcare complications [125].

Table 4 presents an overview of key non-pharmacological strategies currently used to enhance clinical outcomes and quality of life for patients with OSCC.

## 10. Discussion

The evidence synthesized in this review supports the framing of OLP and OSCC as a single clinical continuum rather than as two unrelated conditions and indicates that non-pharmacological modalities can contribute meaningfully at several points along this axis. Across the OLP literature, photobiomodulation emerges as the most consistently supported intervention, achieving outcomes comparable or superior to topical corticosteroids while maintaining an excellent safety profile, positioning it as a credible alternative or adjunct, particularly for steroid-resistant or steroid-intolerant patients. High-power ablative lasers and photodynamic therapy provide additional options for refractory or localized disease, whereas nutraceutical and phytotherapeutic agents, probiotics, ozone therapy, cryotherapy, and psychological interventions remain biologically rationalized but evidentially immature, with efficacy signals frequently confounded by small samples, heterogeneous protocols, and the well-documented tendency of OLP toward spontaneous remission. A recurring and clinically important theme is the unresolved safety question surrounding light-based therapies in potentially malignant and malignant tissue, where the same proliferative stimulus that promotes healing could theoretically favor neoplastic progression; current in vitro and in vivo data are reassuring for standard mucositis protocols but remain strongly parameter-dependent and insufficient for definitive clinical guidance. In the OSCC setting, non-pharmacological measures are best understood as supportive rather than curative, reducing treatment-related toxicity and improving functional and quality-of-life outcomes when integrated into multidisciplinary care. Taken together, these observations indicate that the principal barrier to translation is not biological plausibility but the quality and comparability of the underlying evidence, which must be addressed before firm, modality-specific recommendations can be made.

### Limitations

Several limitations inherent to both the narrative review methodology and the primary evidence base must be acknowledged. As a narrative review rather than a systematic review, the selection of studies reflects the authors’ clinical judgment and may be subject to selection bias, despite the broad database search described above. The primary literature on non-pharmacological interventions for OLP is itself heterogeneous in ways that limit conclusions across all modalities reviewed: sample sizes are predominantly small, follow-up periods rarely exceed 6 months, and outcome measures are inconsistently defined across studies, with some using disease-specific scores (Thongprasom, REU, ABSIS) and others relying on VAS pain score alone. Blinding is rarely feasible for device-based or physical therapies such as PBM and ozone, introducing performance and detection bias that is particularly consequential when the primary endpoint is a subjective pain score; this is not a correctable limitation of individual trials but a structural feature of these intervention categories that constrains the certainty of conclusions. Placebo-controlled data are absent or limited to a single trial for several agents reviewed, including ozone therapy and all coconut-based formulations, making it impossible to rule out a substantial placebo effect in their reported positive outcomes, a concern amplified by the well-documented tendency of OLP toward spontaneous remission. Diagnostic criteria applied across the included studies varied, ranging from clinical-only to histopathologically confirmed OLP meeting the modified WHO criteria, with implications for the comparability of results across studies. Finally, publication bias cannot be excluded, as most available trials report some degree of clinical improvement with the investigated intervention, and null or negative studies may be underrepresented in the accessible literature.

## 11. Conclusions

- Non-pharmacological interventions are increasingly relevant across the OLP–OSCC continuum as complementary, not substitutive, strategies within multidisciplinary care.
- For OLP, photobiomodulation (PBM) currently has the most robust and consistent clinical evidence, with efficacy comparable or superior to topical corticosteroids and an excellent safety profile.
- High-power laser therapy and photodynamic therapy show promising results, particularly in refractory cases, but require larger-scale validation.
- Nutraceuticals/phytotherapeutics, probiotics, ozone therapy, cryotherapy, and psychological support are biologically plausible and well tolerated, but the current evidence is insufficient to recommend them as standalone standard treatments.
- For OSCC, surgery and radiotherapy remain the mainstays, while supportive measures (oral hygiene programs, PBM for treatment-related toxicity, rehabilitation, nutritional and speech/swallowing support) are essential to reduce side effects and improve functional recovery and quality of life.
- Early diagnosis, rigorous long-term follow-up of OLP, and prompt multidisciplinary management are key to reducing progression and the associated healthcare and socioeconomic burden.
- OLP and OSCC should be regarded not as two separate entities but as a continuous disease spectrum, in which early detection, monitoring, and evidence-based non-pharmacological interventions may improve long-term outcomes beyond symptom control.

### Future Perspectives

Addressing the evidence gaps identified in this review will require a coordinated research agenda across several domains. The highest priority is to conduct adequately powered, multicenter, randomized controlled trials with pre-registered protocols, consistent diagnostic criteria (histopathological confirmation according to the modified 2003 WHO criteria as a minimum standard), validated composite outcome measures combining pain, lesion severity, and quality of life, and follow-up of at least 12 months to capture the relapsing–remitting course of OLP. For PBM, which currently presents the most consistent efficacy signal, dose optimization trials are specifically needed to establish the optimal wavelength, power density, energy fluence, and number of sessions, since current evidence favors total energy densities below 120 J/cm^2^ and protocols exceeding ten sessions, but no head-to-head dose comparison RCT has been conducted. For nutraceutical agents, future trials should evaluate nanoformulated or bioavailability-enhanced preparations rather than conventional dose-based systemic regimens, given mechanistic and early clinical evidence that bioavailability, rather than nominal dose, drives efficacy; standardization of the formulation and concentration used across trials is essential to allow meaningful comparisons. For ozone therapy, placebo-controlled trials evaluating ozone monotherapy, rather than the adjunctive ozone-plus-corticosteroid design that currently dominates the literature, would allow its independent contribution to be disentangled from background steroid therapy. Integration of biomarker-based endpoints alongside clinical outcomes, including validated salivary or immunohistochemical markers of inflammation, oxidative stress, and malignant transformation risk (e.g., Ki-67, p53, DNA methylation patterns), would help establish whether non-pharmacological treatments modify disease biology or merely palliate symptoms. Longer-term surveillance studies are also needed to determine whether any intervention meaningfully reduces the risk of malignant transformation in OLP, an endpoint that was not formally assessed in any of the trials reviewed here. Regarding the OSCC continuum, multicenter evaluation of standardized PBM protocols for oral mucositis prevention, using consistent fluence and delivery parameters, would clarify the benefit–risk balance in patients with active or recently treated oral malignancy. Finally, the integration of artificial intelligence tools for early lesion detection and automated clinical scoring, and the development of personalized, risk-stratified treatment algorithms within multidisciplinary oral oncology teams, represent promising research directions that the next generation of clinical trials should incorporate as secondary aims.

## Figures and Tables

**Table 1:** Light-based non-pharmacological therapies for oral lichen planus (OLP).

Therapy	Biological Mechanism	Main Clinical Indication	Main Clinical Outcomes
**High-power ablative laser (CO_2_, Er:YAG, Nd:YAG)**	Photothermal tissue vaporization and ablation	Symptomatic or treatment-resistant OLP	↓ lesion size, ↓ pain, ↓ recurrence
**Low-Level Laser Therapy (LLLT)/Photobiomodulation (PBM)**	Mitochondrial activation, ↓ NF-κB signaling, ↓ TNF-α, IL-1β and IL-6, ↑ tissue repair	Symptomatic OLP	↓ pain, ↓ inflammation, improved healing
**Photodynamic Therapy (PDT)**	ROS-mediated apoptosis of inflammatory cells	Symptomatic OLP	↓ lesion severity, ↓ pain, improved QoL

↑, increase; ↓, decrease.

**Table 2:** Nutraceutical and phytotherapeutic interventions for oral lichen planus (OLP).

Therapy	Main Biological Target	Main Findings	Main Limitations
**Curcumin**	NF-κB inhibition, Nrf2 activation	Anti-inflammatory activity; inconsistent clinical efficacy	Poor bioavailability
**Aloe vera**	Anti-inflammatory and wound healing	Safe; heterogeneous efficacy	Conflicting RCTs
**Coconut-based formulations**	Antioxidant and tissue repair	Promising topical adjunct	Few clinical studies
**Melatonin**	NF-κB modulation, antioxidant activity	Experimental adjunctive therapy	Mainly mechanistic evidence
**Flaxseed**	Cytoprotection	Preclinical evidence only	No clinical trials
**Zinc supplementation**	Immunomodulation	Reduction in pain and lesion severity	Small RCTs
**Vitamin D supplementation**	Immunomodulation	Adjunctive benefit	Small studies
**Other topical formulations (hyaluronic acid, oligomeric proanthocyanidins, umbelliferone)**	Tissue repair	Preliminary positive findings	Limited evidence

**Table 3:** Adjunctive non-pharmacological approaches for oral lichen planus (OLP).

Therapy	Main Mechanism	Clinical Objective	Main Limitation
**Cryotherapy**	Controlled tissue destruction	Lesion reduction	Few clinical studies
**Ozone therapy**	Antioxidant, Nrf2 activation, immunomodulation	Adjunctive therapy	Weak evidence as monotherapy
**Probiotics**	Oral microbiome modulation	Reduction in inflammation	Heterogeneous strains
**Psychological and behavioral interventions**	Stress reduction	Improve symptoms and quality of life	Few controlled studies

**Table 4:** Non-pharmacological management of OSCC.

Intervention	Main Objective	Main Clinical Benefit	Main Limitation
**Surgery**	Complete tumor excision (R0)	Curative treatment	Functional sequelae
**Radiotherapy**	Local tumor control	Improved local control	Xerostomia, dysphagia
**Photobiomodulation**	Management of treatment-related toxicity	Prevention/treatment of oral mucositis	Lack of standardized protocols
**Oral hygiene management**	Prevention of oral complications	Reduced mucositis, infections, osteoradionecrosis	No standardized protocol
**Post-treatment rehabilitation**	Functional recovery	Improved speech, swallowing, nutrition and QoL	Requires multidisciplinary team

## Data Availability

No new data were created or analyzed in this study. Data sharing is not applicable to this article.
